# Confined System Analysis of a Predator-Prey Minimalistic Model

**DOI:** 10.1038/s41598-019-47603-9

**Published:** 2019-08-02

**Authors:** Siddhant Mohapatra, Pallab Sinha Mahapatra

**Affiliations:** 1 Department of Mechanical Engineering, National Institute of Technology Silchar, Silchar, India; 2 Department of Mechanical Engineering, Indian Institute of Technology Madras, Chennai, India

**Keywords:** Mechanical engineering, Applied mathematics, Fluid dynamics

## Abstract

In nature exists a properly defined food chain- an order of hunting and getting hunted. One such hunter-hunted pair is considered in this context and coordinated escape manoeuvres in response to predation is studied in case of a rarely examined confined system. Both the predator agent and prey agents are considered to be self-propelled particles moving in a viscous fluid. The state of motility when alive and passivity on death has been accounted for. A novel individual-based combination of Vicsek model and Boids flocking model is used for defining the self-propelling action and inter-agent interactions. The regimes observed at differing levels of co-ordination segregated by quantification of global order parameter are found to be in agreement with the extant literature. This study strives to understand the penalty on the collective motion due to the restraints employed by the rigid walls of the confinement and the predator’s hunting tactics. The success of any escape manoeuvre is dependent on the rate of information transfer and the strength of the agitation at the source of the manoeuvre. The rate of information transfer is studied as a function of co-ordination and the size of the influence zone and the source strength is studied as a function of escape acceleration activated on the agitated prey. The role of these factors in affecting survival rate of prey is given due coverage.

## Introduction

Darwin’s theory of evolution speaks of adaptability of organisms to adverse conditions in order to ensure survival. One such scenario is that of the presence of a predating species in the vicinity. This adversity might have been a possible reason for organisms to develop co-ordination, which has been termed as collective behaviour or collective consciousness. Such behaviour can be observed all around in nature from human beings to fish to even micro-organisms.

The physics behind this behaviour, however, is highly debatable with research ranging from experimental observations on a flock of birds or a shoal of fish to numerical work under a multitude of conditions^1–4^. Breder^5^ in his widely acclaimed book on collective motion in fish wrote of a possible concept of a leader fish, which has more enterprise or vision than the other fish in the shoal and asserted that the behaviour of this leader fish decides the behaviour of the shoal. Later, Radakov^1^ coined the terms “waves of agitation” and “streams of agitation”, which were used to describe how Breder’s leader fish was able to transmit information to the rest of the shoal. This leadership concept was extended by Rands *et al*.^6^, Couzin *et al*.^7^ and Nagy *et al*.^8^. The different types of manoeuvres conducted by prey aggregates were scrutinized and classified by Pitcher & Wyche^9^. They spoke of eight evasive actions undertaken by the sand-eels, which was later expanded to twelve by Pitcher & Magurran^10^, and compared the probabilities of selection of such actions with variation in surrounding conditions.

As for the numerical and analytical works, a number of models have been developed, where the organisms are considered to be self-propelling particles following certain interaction protocol. The most popular among them are the Vicsek model^11^, Boids Flocking Model^12^ and the more recent Cucker-Smale Model^13^. Numerous additions and enhancements have also been done on these models to make them more veritable^14–18^. Some examples of this can be escape and hunting accelerations in the Boids model and noise in the Vicsek model. While the features of the Vicsek model and the Boids model can be used for only collective behaviour in organisms^19,20^, the Cucker-Smale model is highly diverse, finding use in finance and other dissimilar fields^21^. The Cucker-Smale model has also undergone some modifications by addition of noise^22^ and incorporation of repelling force^23^. Apart from these models, some models using only attractive-repulsive forces^24–26^ or using Morse potential and Leonard-James potential alongside alignment conditions^27^ have also been proposed. Fuzzy logic approaches, apart from its diverse use in taxonomy^28^ and logistics & crowd optimization^29^, have also been used more than often especially in developing genetic algorithms for evolutionary studies^30^. These evolutionary models, emerging of late have provided some tremendous insight in deciphering the predator-prey relation. Nishimura^31^ discussed possible tactics (based on priority functions) which a predator might use to attack: nearest prey, peripheral prey or isolated prey. This line of study has been bolstered by works of Demšar & Bajec^32^ and Demšar *et al*.^33^. Using Markov network analysis, Olson *et al*.^34^ concluded that the evolution of collective behaviour for flight response improves in case of density dependent predatory attacks. Change in predatory tactics is a critical factor for the evolution of swarming behaviour as reported by Demšar *et al*.^35^. However, the origin of the collective behaviour as is seen in nature lies shrouded in mystery. Biswas *et al*.^36^ promotes the theory of dilution of risk with increasing prey numbers, which instigated the prey individuals to stick together. At the same time, predator confusion has also been cited as a possible reason for the development of this co-ordination, as mentioned by Olson *et al*.^37^.

Although a plethora of models and investigations exist on this topic, a large part of it is based on open-domain conditions^3,4,38–41^ or periodic boundary settings^18,42^. Existing closed domain works like that of Olson *et al*.^34,37^, Demšar *et al*.^32,33,35^ and Hein *et al*.^43^ focus on the evolution of predation and flight tactics or strive to uncover the origins of collective behaviour. These works don’t provide much information on the effect of confinement on collective behaviour. Some closed domain works like that of Marras *et al*.^44^ and Gautrais *et al*.^45^ deal with the interaction with the wall, however, they have not included the predatory element in the system. Hence, there is a need for further research on the predator-prey system in a limited space. The current work aims to thoroughly study the response of the prey to the presence and actions of a predator within a confinement. To this end, a novel zone-based combination of Vicsek and Boids model has been developed. The effect of co-ordination level of the agents as well as the escape acceleration of the agitated prey have been studied in detail. Order parameter of a flock, which denotes the sense of ordering in a flock, has always been studied in much detail, be it for a system of only motile particles or a system of mixture of self-motile and passive particles^46^. The order parameter along with the survival rate provides a quantification of the ramifications of co-ordination and escape acceleration in the successful occurrence of flight manoeuvres. The outcome of the presence of the confinement and the presence of dead agents have been thoroughly discussed in the purview of order parameter.

## Results

### Formation of regimes

**Figure 1:**
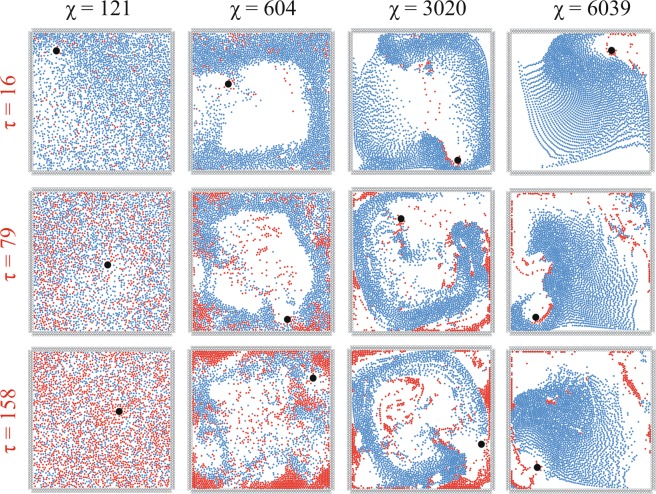
Recurring behaviour of agents is shown at different instances of non-dimensional time $\tau =t/\sqrt{\frac{L}{\beta }}$ under changing conditions of non-dimensional co-ordination co-efficient $\chi =\frac{{C}_{v}L\sqrt{L}}{m\sqrt{\beta }}$. At $\chi =121$, thermal motion of the agents takes place, which develops into a milling (vortical motion with an empty core) regime at $\chi =604$. At $\chi =3020$, the intensity of milling has decreased and due to decrease in the strength of the centrifugal force on the agents, the unfilled core keeps reducing in size as *τ* increases. At $\chi =6039$, motion features pertaining to the dynamic parallel grouping (oscillatory regime) is observed alongside remnants of vortical motion. (Note: The initial position of the predator is (0.93, 0.43) in a 1 × 1 domain. Images shown are representative of the usual trend observed over all the trial cases. Blue, red and black represent live prey, dead prey and the predator respectively).

In order to study the predator-prey relationship, simulations are carried out on a number of prey agents and a singular predator in a closed square box under a variety of circumstances. One of the principal parameters used in this regard is that of *χ*, which represents the relative importance of coordination force over the self-propelled force, i.e. higher co-ordination for the same amount of self propulsion corresponds to higher value of *χ*. The behaviour and interactions of the agents are observed and analysed for an appreciable range of *χ* (see Fig. 1). At very low values of *χ*, thermal motion of agents is observed due to lack of the co-ordination force. The case $\chi =121$ has the highest predator kill-rate among all the values of *χ* tested. This pertains to the fact that the amplitude of motion of an individual prey agent is smaller than that of the predator owing to the lesser mass of the prey. Due to lower co-ordination forces, the agents are able to move with higher individuality.

At lower intermediate values of *χ* (like $\chi =604$), the agents display a distinct vortical motion around a completely vacant core (called milling^47^). This milling motion of the agents is, on many a occasion, disrupted by the movement of the predator. Especially when faced with the predator and the confinement boundary on either side, the agents disperse due to co-ordination being on the lower side, instead of performing a cohesive escape manoeuvre. The dispersed agents reassemble after the escape force stops acting (i.e. when the predator is no longer within their detection zone). Another point of note is the accumulation of dead agents at the corners of the square confinement. The reason behind the entrapment is the lack of self-propulsion force of dead agents due to which they are pushed to the sides of the flocks during the motion, and eventually get stuck in these corners. Some agents stay suspended in the unfilled core section as centrifugal force no longer acts on these inanimate agents, which are now away from the flock and have also lost the ability to self-propel.

With increasing values of *χ*, the empty core gets smaller and easier to disrupt. At $\chi =3020$, the motion of the agents still has a milling element, but the intensity of centrifugal force acting on the agents has decreased, hence, the size of the unfilled core has become variable, with agents passing through its midst in lieu of an escape manoeuvre. At higher values of *τ*, the degree of disruption is enhanced. The escape mechanism is also more prominent in this case as the co-ordination force is much higher than the self-propelled force. As a result, the escape force is well coordinated with the neighbours and the escape manoeuvre is executed better. The predator having higher mass also faces a greater resistance towards movement making the hunt more prone to failure.

At even higher values of *χ*, only minute traces of the vortical motion remain and the motion is now dynamically parallel^47^ in nature. This kind of motion is due to the position restraints imposed on the agents. If there were no boundaries, the prey would totally elude the predator, as can be seen in Supplementary Information I. However, in this case, the prey on encountering the walls of the confinement are repulsed and aligned along the wall for an instant of time. This is a striking consequence of using a confined space, which is covered in greater detail in Supplementary Information I. As the co-ordination force acts like a friction force for the predator, at $\chi =6039$ (at high co-ordination force), the friction force acting on the predator is the highest among all the *χ* values tested. This results in the retardation of the predator’s movement causing the dead agents to fill up the entire detection range of the predator, rendering the predator unable to kill. Hence, there is a sharp decrease in the number of dead agents.

These results are in good agreement with existing literature^42,48,49^ regarding the improvement in escape manoeuvre and collective motion with increase in co-ordination (increase in *χ*). With increase in grouping behaviour, the survival instincts kick in better, while hunting is inhibited by a stronger resisting force. Hence, there is a perceptible decrease in number of dead prey with increase in *χ* (see Fig. 1). For further understanding, related videos can be found in Supplementary Video I.

### Order parameter

The global order parameter *ϕ* has been defined in many earlier texts^2,38,50–52^ to describe the flocking sense of the agents in consideration. In the current work, the non-dimensional polarization defined by Cavagna *et al*.^38^ has been used.

1 $$\varphi =|\frac{1}{n}\sum _{i=1}^{n}\frac{{\overrightarrow{v}}_{i}}{|{\overrightarrow{v}}_{i}|}|$$

where, ${\overrightarrow{v}}_{i}$ represents current velocity of *i*^*th*^ agent and *n* is the total number of prey agents.

**Figure 2:**
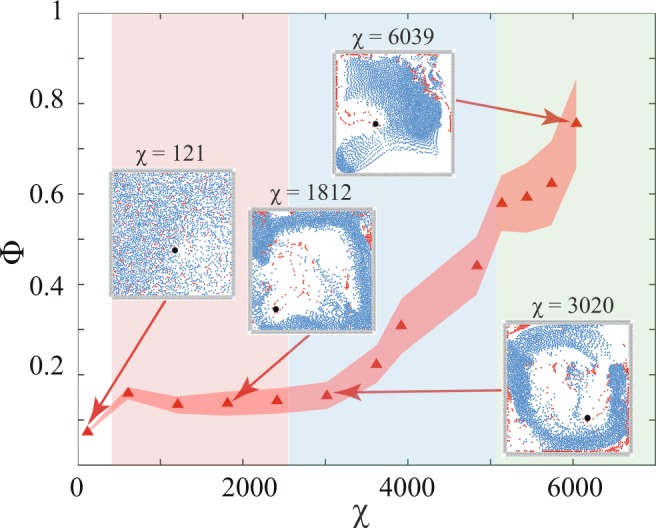
The temporally averaged order parameter Φ is plotted for increasing *χ* values. The entire plot can be approximated into 3 distinct regimes: (i) $\chi =604\to 2416$ characterized by a vortical motion (highlighted in red) (ii) $\chi =2416\to 5133$ characterized by a transition from milling to dynamically parallel motion (highlighted in blue) (iii) $\chi =5133\to 6039$ characterized by a dynamically parallel motion (highlighted in green) (Note: Representative snapshots of the different regimes are shown in the inset.  denotes the average Φ values over multiple initial positions of the predator and the shaded band denotes the standard deviation for the same with 95% confidence interval. The statistical trend is further explained in Supplementary Information II. The non-highlighted part consisting of $\chi =121$ shows thermal motion and has been excluded while defining regimes as it doesn’t involve coordinated behaviour).

Following existing research on behaviour of the collective system, the temporally averaged order parameter Φ should increase with increase in co-ordination among the agents (increase in *χ*)^2,51,53^. Mahapatra & Mathew^53^ used a very similar method of ascertaining the polarization of the congregating particles and observed increasing alignment when *χ* increased. A similar trend is observed in the current work, i.e. the temporally averaged ordering of the flock increases with increasing *χ* (see Fig. 2). Φ yields three distinct regimes when plotted against *χ* (see Fig. 2) as has already been shown in Fig. 1. The first regime is characterized by milling motion of the agents, where the instantaneous alignment of velocities of all the revolving agents is not achieved. As *χ* increases, the coherence of the agents keeps strengthening (see Fig. 2 inset at $\chi =1812$). The second distinct regime is a transitional regime consisting of both vortical as well as dynamically parallel motion components (see Fig. 2 inset for $\chi =3020$). Ability to herd is much better. In the first two regimes, due to the presence of the vortical component of motion, the dead agents get pushed to the boundaries of the confinement. The third distinct regime, however, comprises of almost entirely oscillatory behaviour and herding behaviour is further improved as only a minuscule vortical component remains (see Fig. 2 inset for $\chi =6039$) and dead agents do not accumulate at the edges of the confinement. The non-highlighted part in Fig. 2 comprising of $\chi =121$ has been excluded from the regime classification. This is due to very low values of coordination force leading to thermal motion of the agents, which is an uncoordinated behaviour.

**Figure 3:**
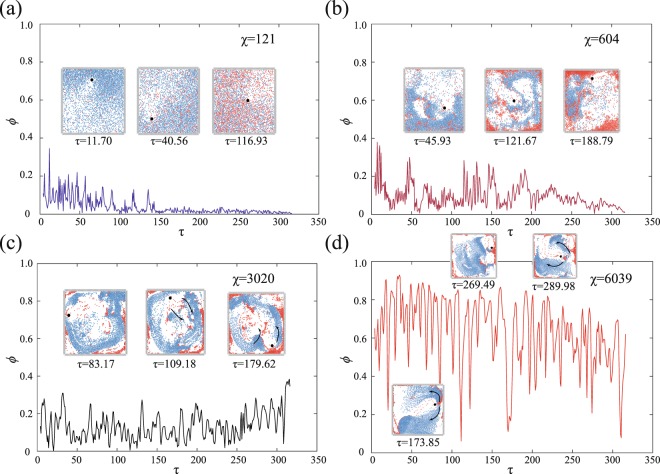
The variation of order parameter *ϕ* with non-dimensional time *τ* is shown for regime-specific *χ* values. The inset images show the state of the system at different instances of simulation time. The order parameter fluctuations increase as *χ* increases due to enhanced escape manoeuvres (like, split and join and fountain) and reduced predation capability due to a stronger inhibitory force on the predator. The manoeuvres are graphically elucidated in Supplementary Video II (Note: Predator’s initial position is (0.93, 0.43) in a 1 × 1 domain. This image is representative of all the trial cases with respect to initial position dependency of predator. $\chi =121$ is shown in order to emphasize the occurrence of thermal motion).

The polarization of the flock, described in Eq. 1, when observed against the time scale, yields an increasingly fluctuating curve for increasing values of *χ* (see Fig. 3). There are two reasons for these fluctuations: “predatory influence” and “effect of confinement”. The minor fluctuations can be attributed to the effect of confinement, while the major dips and spikes are the result of predation. At higher values of *χ*, as the flock approaches the wall of the confinement, the front-line agents experience a repulsion and subsequent temporary alignment along the wall. This sudden change in direction of these agents causes confusion in the flock and hence the directions of the individual agents, which were well-aligned earlier, go awry. This sudden dis-alignment in the flock causes a small dip in the value of polarization. However, the effect of the repulsion is short-lived as the flock again starts moving in a weighted mean direction and this re-alignment restores the polarization of the flock. The said repulsion can be observed in Supplementary Videos I and II. However, the same can not be said for the vast fluctuations occurring a few times at $\chi =604$, $\chi =3020$ and $\chi =6039$. At $\chi =604$, the predator disrupts the motion of the agents, distorting the milling core as can be seen in the Fig. 3(b) insets. This is continued at $\chi =3020$. As the strength of the vortical regime decreases, the prey are able to move right through the core of the milling structure. This in turn allows the prey to perform the split and join evasive manoeuvre as seen in the $\tau =109.18$ and $\tau =179.62$ insets of Fig. 3(c). On approach of the predator, the flock splits into different parts to escape the predator’s vicinity and regroup at a later time. This causes the heavy fluctuations observed in this plot (see Fig. 3(c)). The situation becomes more dire for the predator at $\chi =6039$, when with increased co-ordination, the efficiency of the evasive manoeuvre also increases. On the approach of the predator, the flock splits completely into two divisions and moves in opposite directions joining beyond the tail of the predator (see insets $\tau =173.85$ and $\tau =289.98$ in Fig. 3(d)). This escape manoeuvre is similar to the fountain manoeuvre coined by Pitcher^54^ and refined by Hall *et al*.^55^. As a result, the multiple aggregations of the velocities being opposed in direction, causes a large decrease in the value of polarization. This is responsible for the rapid diminution of the plot at some points (see Fig. 3(d)). Supplementary Video II provides graphic aid in understanding the reason behind these fluctuations.

**Figure 4:**
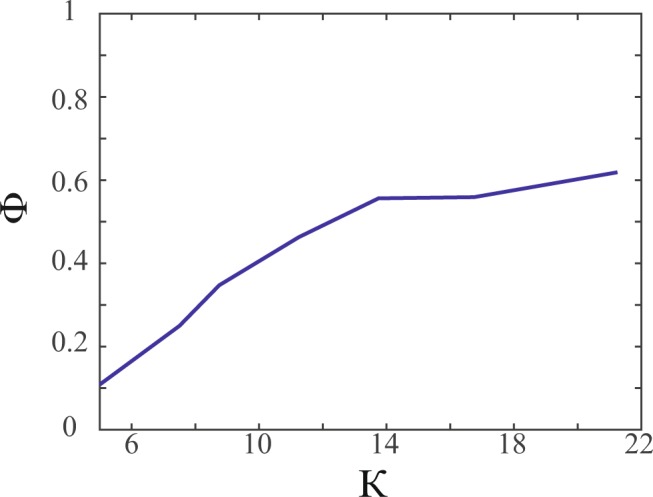
The variation of temporally averaged order parameter Φ is shown against non-dimensional radius of influence *κ* (ratio of radius of influence to the diameter of agent) for $\chi =604$. Φ shows an increasing trend with increase in *κ*, as the number of interacting neighbours increase, which in turn, leads to orientation of more neighbours in the general direction of the now larger local flock (Note: Predator’s initial position is (0.93, 0.43) in a 1 × 1 domain. Similar behaviour is obtained for other positions as well as different *χ* values).

Order parameter also provides us an idea regarding the rate of information transfer in the flock, which is intricately related to flight success. In order to study the extent of the information transfer, the temporally averaged polarization Φ has been plotted against the radius of the influence zone (the area in which the influence of the agent is experienced). A parameter *κ* is used as an indication of the size of the influence zone and is defined as the ratio of the radius of influence zone to the diameter of the circular agent. Φ is observed to have an increasing trend with increase in *κ* (see Fig. 4). This trend is similar to that reported by Couzin *et al*.^47^. As the area of the zone increases in this confined scenario, the number of agents neighbouring each agent increases. This, in turn, causes velocity vectors from a larger local flock to be considered while computing the general direction of the flock. The members of this larger flock are acted upon by a co-ordination force which adjusts the headings of the individual members to match the general direction. As the co-ordination is improved, the value of order parameter increases and at the same time, the flight tactics are carried out more effectively as has been explained in the next section.

### Effect of escape acceleration on escape success

**Figure 5:**
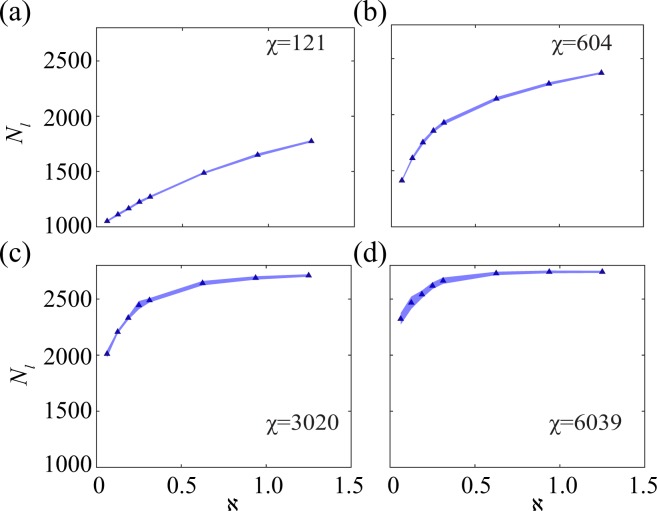
The number of live agents *N*_*l*_ at end of simulation time has been plotted against non-dimensional parameter **ℵ** (the ratio of escape force to hunting force) at regime-specific values of *χ*. The survival rate of the prey increases with increase in **ℵ**. This is true for all the *χ* values presented. *N*_*l*_ increases at a decreasing pace as *χ* increases, i.e. the effect of a higher escape acceleration diminishes as *χ* increases. At $\chi =6039$, negligible change in *N*_*l*_ with increase in **ℵ** is observed. (Note:  represents the average *N*_*l*_ values considering the multiple initial positions of the predator and the shaded bands denote the standard deviation of the same with 95% confidence interval. The statistical trend is further elucidated in Supplementary Information II).

From existing literature^1,9^, it is surmised that escape capability of the prey agents should be highly dependent on the escape acceleration. In order to explore whether domain dependence is a factor in the relation between survival and escape acceleration, the number of live agents at the end of the simulation (in a closed domain) has been plotted against escape acceleration for different co-ordination levels (see Fig. 5). There is a prominent surge in the survival rate with increase in *χ*. This is quite obvious as the increased co-ordination leads to execution of more successful flight manoeuvres. The number of live prey at end of simulation time (*N*_*l*_) also increases with increase in **ℵ** (defined as ratio of escape to hunting forces) for different *χ* values presented. At $\chi =121,604$ and 3020, the curves have an increasing nature (see Fig. 5(a–c) respectively). The mechanism of any escape manoeuvre involves detection of the predator by a small number of prey agents followed by the activation of their escape force and transfer of the predator information to the rest of the flock through the inter-agent interaction force. As can be seen in Supplementary Video I, the prey agents stick together in a tight flock in case of $\chi =3020$, while no such co-ordination is observed at $\chi =121$. Hence, the process of transmission is faster in $\chi =3020$ as there are more number of neighbours in the vicinity of the agitated prey due to higher co-ordination. On the other hand, at $\chi =121$, due to less number of neighbours in the immediate vicinity of the agitated prey, the information transfer takes place rather poorly compelling the agitated prey to try to evade the predator by themselves, and not through elaborate group manoeuvres as is the case at $\chi =3020$. As *χ* increases, the plot of the survival rate of the prey agents shown in Fig. 5 becomes less steep with increase in **ℵ**. At $\chi =6039$, for higher values of **ℵ**, the curve shows negligible change (see Fig. 5(d)). This signifies that the level of co-ordination affects the escape success of the prey to a larger extent than the escape acceleration as the co-ordination of the prey improves.

**Figure 6:**
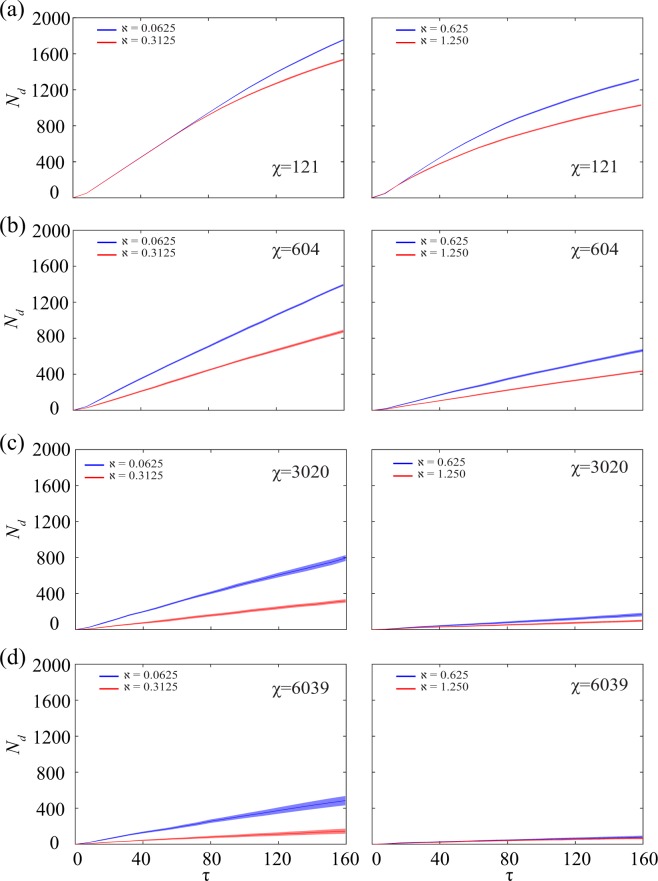
The number of dead agents *N*_*d*_ has been plotted against non-dimensional time *τ* for different values of *χ* and **ℵ**, averaged over all twenty initial positions of predator. The curves are smooth and can be approximated with sufficient accuracy as straight lines. The slope of these curves decreases as *χ* increases, indicating lower deaths due to better flight manoeuvres. The solid lines represent the mean of the *N*_*d*_ values and the shaded bands portray the standard deviation with 95% confidence interval.

To study the predation rate throughout the simulation time, the number of dead prey *N*_*d*_ is plotted against non-dimensional time *τ*. As can be seen in Fig. 6, the results yield smooth mean value curves indicating an invariability in the hunting procedure of the predator. These curves, with constant slope (i.e. straight lines), are in the same vein with the previous results (see Fig. 5) as the number of dead prey agents *N*_*d*_ is higher for lower *χ* (lower co-ordination). With increasing **ℵ** values, the slope of the mean curve is found to be decreasing. The shaded bands provided about each solid curve represents the standard deviation (with 95% confidence interval) of the *N*_*d*_ values across the twenty initial predator positions. All the curves are found to have minute standard deviation values, which denotes low dependence on predator’s initial position.

All the aforementioned results are based around a non-consuming predator (i.e. the prey agents are still part of the system even after death). However, as can be noted from previous explanations, the presence of the dead prey agents in the simulation also affect the predator as well as the live prey’s performance. To shed some light on this conjecture, a few simulations have been run using a consuming predator (i.e. the prey agents are immediately removed from the simulations on death) and the results are shown in Supplementary Information III and Supplementary Video IV.

## Discussion

The interplay amongst conflicting species is an interesting aspect of nature, which has been under extensive scrutiny since the last few decades. However, many a avenue remain unexplored in this domain of research. One such line of thought is that of the effect of the boundary conditions on the predator-prey system. In works of Pitcher & Wyche^9^, Pitcher & Magurran^10^, Thiebault *et al*.^39^, Gerlotto *et al*.^40^ and so on, which are all experiments in open domain conditions, there is more freedom of movement with respect to space. As a result, the behaviour of the predator and the manoeuvres of the prey are unbridled. Periodic domain works by Grégoire & Chaté^18^, Mateo *et al*.^42^ and so on, also provide a semblance of freedom of space, even if it is based on assumptions. Speaking of a closed domain system, Marras *et al*.^44^ conducted an experiment on the rate of information transfer and startle response of fish to an acoustic perturbation in a tank. However, the effect of the boundary conditions on the system was not clarified. A different study by Gautrais *et al*.^45^ concluded that the response of a fish is a function of its distance from the wall. Although, the disparity in behavioural patterns of fish in a confined domain as compared to an open domain was established, no predator was considered in the study, and hence, no conflict took place. The more recent works in the closed domain such as those by Demšar & Bajec^32^, Demšar *et al*.^33,35^, Olson *et al*.^34,37^ either strive to understand the evolution of predation and flight tactics or focus on the origin of collective behaviour and consider the confinement as only a convenient simulation boundary. The effect of the presence of this confinement on the prey flight tactics is not discussed in these works.

In the current study, collective flight manoeuvres of the prey in evading a predator has been studied with specific emphasis on elucidating the ramifications of the limited space for evasion. The collective behaviour of the prey as observed from our results is divided into three distinct phenomenological regimes, depending on the level of co-ordination among the prey. A scattered, less interactive behaviour is seen at lower *χ* values, which steadily strengthens to a highly interactive behaviour as *χ* increases. This leads to a marked improvement in success of escape strategies with increase in *χ*. An order parameter is implemented to provide a value to the level of interaction in the flock. The temporally averaged order parameter is computed to quantitatively distinguish among the three regimes. A temporal study of polarization yields fluctuating plots most of which are attributed to the predatory influence, which kicks off elaborate escape manoeuvres like that of split and join and fountain. The effect of confinement also has a role in play in these fluctuations, resulting in a momentary misalignment in the headings of the prey agents. A more detailed analysis of the effect of confinement is discussed in Supplementary Information I. The relation of order parameter to the increase in *χ* has already been established. However, *χ* is not the only factor that co-ordination depends upon. The radius of influence in a metric distance based interaction model plays an important role too. As this radius is increased, the co-ordination also increases, as portrayed by the rise in the order parameter. Increase in co-ordination can be interpreted as an increase in the rate of information transfer made possible by improvement in flocking behaviour (milling to dynamically parallel grouping). In this work, the information transfer takes places in the form of force, rather than signal. The increase in the number of neighbours is seen to have positive effect on the escape success, as the predator-related information is transferred to a larger section of the flock at a time, instead of being subject to multiple transfers, in which case, the force containing the information undergoes continuous attenuation. On conducting a parametric study, the escape success of the prey is found to depend on the escape acceleration of the prey. When observed alongside co-ordination, the escape acceleration has a crucial role in boosting the survival rate of the prey. A successful group escape action depends on how quickly the information regarding the approach of the predator reaches out to the nearby prey agents. This, in turn, depends on the source strength of the agitation (in our work, the escape force) and as discussed earlier, the number of neighbours in the vicinity. If the agitation at source is stronger, it can travel a longer distance through multiple interactions without significant attenuation. In order to quantify how the source strength of the agitation affects the escape success, the escape acceleration is increased by increasing **ℵ** and the corresponding survival rate is computed. The curves are of increasing nature, showing a increase in survival rate with increase in source strength. On comparing the curves at different co-ordination levels (see Fig. 5), higher co-ordination yielded flatter curves, i.e. the increase in source strength (**ℵ**) of the agitation has a less pronounced effect on the survival rate as *χ* increases (for details see the regression trend in Supplementary Information II). A temporal study on the killing rate of the predator ensued in smooth averaged curves, which can be approximated as straight lines without loss of accuracy. These curves connote the absence of major variations in predator activity throughout the simulation time and across different initial positions of the predator. The predatory action has been seen to be affected by both the confinement and the dead agents. The role of the dead agents in inhibition of predation is explained in Supplementary Information III.

While these simulations provide insight into the predator-prey interaction conundrum, it is built on various assumptions and is only a minimalistic model, while corresponding biological processes represented are quite complex in nature. Hence, the scenario can be made more realistic by addition of multiple predators, neutral agents and obstacles or by introducing a food chain by assimilating multiple predator-prey systems and so forth. Self learning algorithms can also be implemented to have the prey learn to avoid the walls instead of the approach and repulse mechanism used in this work. Behavioural artificial intelligence can be integrated into the predator model to have it think of possible outcomes before pursuing any prey agent.

## Methods

### Governing equations

The model considered in this study is a modified Vicsek model^11^ with features incorporated from the extended Reynolds’ Boids flocking model^12^. In this system, there are two different kinds of agents- prey and predator, with the latter pursuing and killing the former. All the prey agents can have two states of existence: active (i.e. alive) and inactive (i.e. dead). The predator is considered to be active throughout the simulation. An active agent has the ability to propel itself as long as it is alive.

In this study it is considered that, each agent can be subject to inter-agent interaction force ${\overrightarrow{F}}_{pp}$, self-propulsion force ${\overrightarrow{F}}_{sp}$, co-ordination force ${\overrightarrow{F}}_{c}$, hunting force ${\overrightarrow{F}}_{h}$ and escape force ${\overrightarrow{F}}_{e}$ depending on the nature of the agent and subject to fulfilment of certain pre-requisites. As described earlier, the self-propelled force and the co-ordination force acts on the active prey agents only, while the hunting force and the escape force acts on the active predator and active agitated prey respectively.

Total force acting on *i*^*th*^ prey agent can be defined as:

2 $$\overrightarrow{F}=\xi {\overrightarrow{F}}_{sp,i}+\xi {\overrightarrow{F}}_{c,i}+{\overrightarrow{F}}_{pp,i}+\xi {\overrightarrow{F}}_{e,i},$$

where *ξ* is the state of living and has values 1 and 0 for live and dead agents respectively.

Total force acting on a predator agent (*p*^*th*^ agent) can be defined as:

3 $$\overrightarrow{F}={\overrightarrow{F}}_{sp,P}+{\overrightarrow{F}}_{c,P}+{\overrightarrow{F}}_{pp,P}+{\overrightarrow{F}}_{h,P}.$$

Here, *ξ* is absent as the predator is assumed to be alive throughout the simulation.

The self-propelled force on the prey agent ${\overrightarrow{F}}_{sp,i}$ is modelled as,

4 $${\overrightarrow{F}}_{sp,i}={m}_{i}(\beta -\alpha |{\overrightarrow{v}}_{p,i}{|}^{2}){\hat{v}}_{p,i},$$

where *m*_*i*_ is the mass of the prey agent, ${\hat{v}}_{p,i}$ is a unit vector in the direction of the velocity of the *i*^*th*^ agent ${\overrightarrow{v}}_{p,i}$ and *β* is a thrust coefficient. A small parameter *α* is used in Eq. 4 to restrict the unbounded acceleration of the agents. For a dense system, the dependency of *α* on the final solution is very less^56^. Similarly for the predator, the self-propelled force is modelled as,

5 $${\overrightarrow{F}}_{sp,P}=M(\beta -\alpha |{\overrightarrow{v}}_{P}{|}^{2}){\hat{v}}_{P},$$

where *M* is the mass of the predator, ${\hat{v}}_{P}$ is a unit vector in the direction of the velocity of the predator.

The coordination force ${\overrightarrow{F}}_{c,i}$ used here is similar to the alignment force or friction force^57,58^ used in the literature. The motion of the agents interacting through local alignment, is considered. This force actively promotes alignment only in the live prey agents. The dead prey agents are not subject to this force. Similar to the Mahapatra *et al*.^56^ and Carillo *et al*.^58^,

6 $${\overrightarrow{F}}_{c,i}={C}_{v}{d}_{i}({\overrightarrow{v}}_{i}-{\overrightarrow{v}}_{p,i}),$$

where, *C*_*v*_ is a coordination coefficient and ${\overrightarrow{v}}_{i}$ is the average velocity of the agents. This force makes up for the absence of local coordination, and therefore provides the coordinated motion. In the absence of the coordination force or at very low values of the co-ordination force, only thermal motion is observed. The coordination coefficient *C*_*v*_ is designed as a parameter in the simulations and a parametric study has been performed at differing values of *C*_*v*_. For the single predator, the coordination force essentially works like a friction force of the form,

7 $${\overrightarrow{F}}_{c,P}=-\,{C}_{v}{d}_{P}{\overrightarrow{v}}_{P}.$$

where, *d*_*P*_ is the diameter of the predator agent and ${\overrightarrow{v}}_{P}$ is the velocity of the predator.

The inter agents interaction force ${\overrightarrow{F}}_{pp,i}$ are modelled as^59,60^,

8 $${\overrightarrow{F}}_{pp,i}=\{\begin{array}{ll}-{k}_{n}\lambda {\overrightarrow{n}}_{ij}, & \lambda  > 0\\ \overrightarrow{0}, & {\rm{otherwise}}.\end{array}$$

where $\lambda =(|{\overrightarrow{r}}_{i}-{\overrightarrow{r}}_{j}|-(({d}_{i}+{d}_{j})/2))$, is the separation between two agents *i* and *j* in terms of position vectors ${\overrightarrow{r}}_{i}$ and ${\overrightarrow{r}}_{j}$ and diameters *d*_*i*_ and *d*_*j*_ for all neighbouring agents *j*. Here, ${\overrightarrow{n}}_{ij}=\frac{{\overrightarrow{r}}_{i}-{\overrightarrow{r}}_{j}}{|{\overrightarrow{r}}_{i}-{\overrightarrow{r}}_{j}|}$ represents the direction of the line joining the agents. For the prey, this interaction force acts between the agents as well as between the agents and the boundary. For the predator, a similar type of force acts between the predator and the walls.

In Eq. 2, the escape force ${\overrightarrow{F}}_{e,i}$ acting on the prey is modelled as,

9 $${\overrightarrow{F}}_{e,i}={m}_{i}{\beta }_{e}{\hat{x}}_{e,i},$$

where *m*_*i*_ is the mass of the *i*^*th*^ prey agent which fulfils the escape criteria, ${\hat{x}}_{e,i}$ is a unit vector in the shortest distance between the predator and the prey agent in the direction opposite to the position of the predator and *β*_*e*_ is a thrust coefficient.

In Eq. 3, the hunting force ${\overrightarrow{F}}_{h,P}$ acting on the predator is modelled as,

10 $${\overrightarrow{F}}_{h,P}=M({\beta }_{h}-\gamma |{\overrightarrow{v}}_{P}{|}^{2}){\hat{x}}_{h,P},$$

where *M* is the mass of the predator, ${\overrightarrow{v}}_{P}$ is the velocity of the predator, ${\hat{x}}_{h,P}$ is a unit vector in the direction of the shortest distance from the predator towards the target prey particle and *β*_*h*_ is a thrust coefficient. *γ* is the Rayleigh’s friction factor^61^ and is included to ensure that the predator does not have unbounded acceleration, which would lead to unrealistic circumstances due to the high mass of this agent.

### Predator-prey interaction model

**Figure 7:**
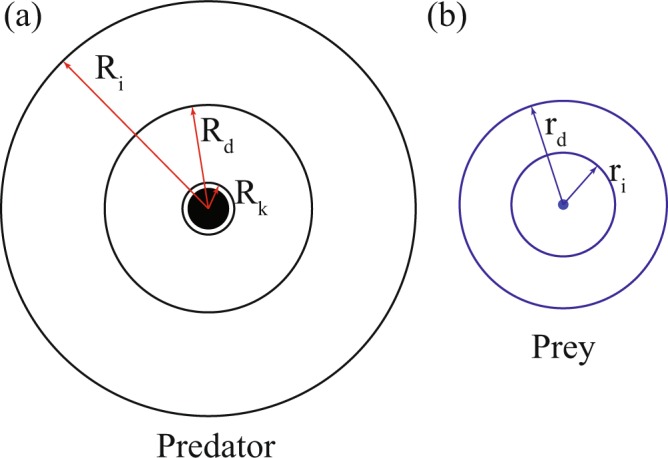
The two different kinds of agents- prey and predator are shown along with their zone-based interaction structure. (**a**) The predator agent has three zones- *R*_*i*_ is the radius of influence zone, *R*_*d*_ is the the radius of detection zone and *R*_*k*_ is the radius of sure-kill zone. (**b**) The prey agent has two zones- *r*_*i*_ is the radius of influence zone and *r*_*d*_ is the radius of detection zone.

Previously, researchers have tried to give different insights into the mechanism of interaction between the predator and the prey agents. However, the definition of predator and prey (see Fig. 7) has remained consistent throughout all these works^3,9,42,62^, except in cases of non-cooperative prey behaviour like cannibalism^63^. The predator is usually defined as a much larger and stronger agent which attacks and consumes prey agents. In the current work, the predator agent has been taken to be only four times as large as the prey agent. Therefore, it is not possible for the predator to attack and consume the prey agent whole like gape predators^64,65^ generally do. Hence, the predator is considered to disturb, pursue, attack and kill the prey agents but is able to feed on only a negligible portion of the prey agent. Therefore, contrary to earlier works, the dead prey agents are not removed from the simulation. Rather, they continue floating in the domain, their motion, depending on the motion of the live agents around them. The live prey agents, on the other hand, are considered to move away from the predator (i.e. conduct an evasive action) in order to ensure their survival. This pursue and evade actions of the predator and prey respectively are brought into effect by the hunting force and the escape force. Figure 7 shows the zone-based model adopted in this work for the agents.

#### Escape action

Experimental studies by Radakov^1^, Breder^5^, Pitcher & Wyche^9^, Handegard *et al*.^66^ and many more have shown the collective response of a swarm of agents to a certain predatory perturbation. In such cases, the agents that are next to the perturbation get agitated and this agitation stream^1^ transmits the information regarding predation to the rest of the swarm. In the present model, the agitation described earlier occurs in form of an escape force, which acts on the select prey agents. The escape force enables the prey agents to evade the predator by aligning its motion in a direction opposite to the position of the predator. However, it doesn’t guarantee successful evasion of the prey agent. The escape manoeuvre of a local prey swarm occurs in three stages: predator detection, escape force update on agitated prey and transmission of predator information to neighbouring prey agents.

**Figure 8:**
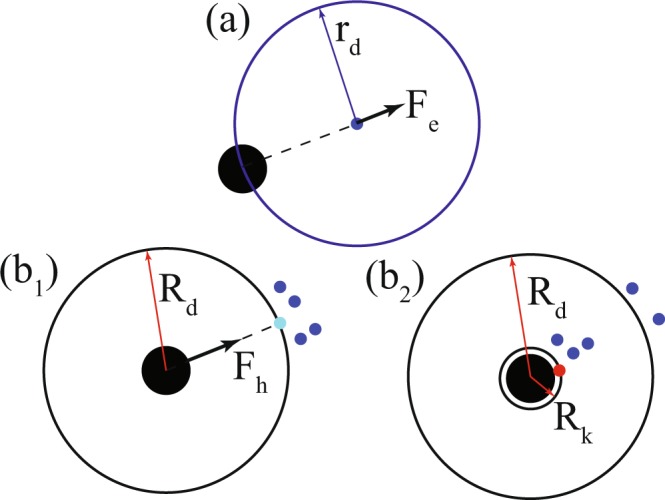
The mechanics of hunting and escape are elucidated along with the technicalities of the satisfaction state. The first part pertains to the prey, while the remaining pertain to the predator. (**a**) Predator detection and escape force ${\overrightarrow{F}}_{e,i}$ update on agitated prey. (b_1_) Prey detection, target selection and hunting force ${\overrightarrow{F}}_{h,P}$ update after waiting. (b_2_) Hunting success followed by satisfaction stage. (Note: Target has been coloured cyan, dead prey red, live prey blue and predator black).

In the first stage, one or more prey agents detect the predator within their detection zone *r*_*d*_. This detection zone is taken to be circular in nature (see Fig. 7(b)). As such, the prey agents are considered to have a 360 field of vision. Upon detection, these agents get agitated and escape force acts on these agents (see Fig. 8(a)). This force acting on these few agitated agents is transmitted to their immediate neighbours, i.e. the agents within their radii of influence *r*_*i*_, through the inter-agent interaction force. These neighbours then transmit the force to their neighbours and a cascading effect is obtained in form of an evasive manoeuvre. Furthermore, in cases where multiple number of agents detect the predator and have escape forces in different directions, the direction of motion of the swarm is decided by aligning the velocity vector of the swarm along the mean weighted velocity of all the prey agents in the swarm. This is achieved by the co-ordination force ${\overrightarrow{F}}_{c}$, which has been described earlier. Once the escape is successful, i.e. the prey confirms that the predator is no more in its vicinity, the escape force stops acting on the prey agent.

#### Predation

In the current work, the predator agent is defined as a moving source of perturbation in the system of agents, which pursues and kills prey agents. Nevertheless, these actions occurs in a pre-set sequence and subject to certain terms and conditions. Following works by Demšar *et al*.^33^ and Mateo *et al*.^42^, the predator agent described in this model also adheres to a certain set of rules while hunting. In this model, predation takes place by application of a hunting force ${\overrightarrow{F}}_{h}$ on the predator. This force acts in a direction along the shortest distance from the predator towards the target prey.

**Figure 9:**
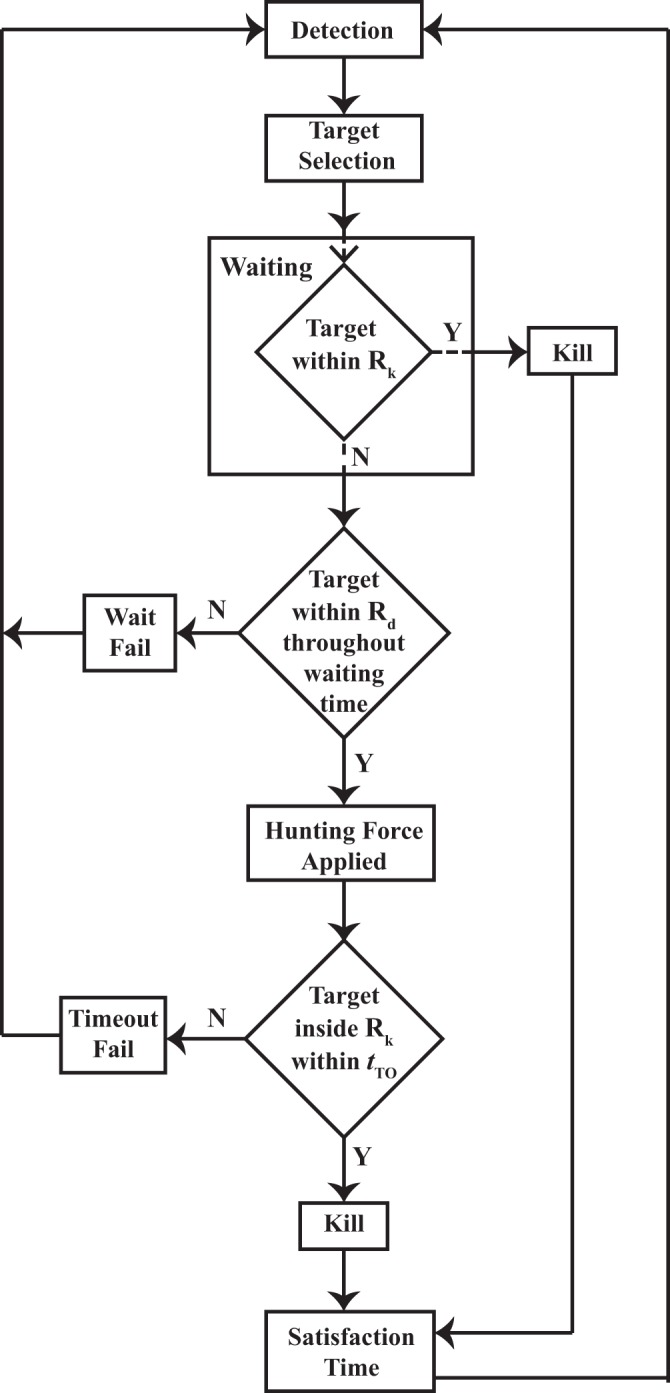
The algorithm governing the predation mechanism has been illustrated through a flowchart. The different stages of the mechanism are: (i) Detection & Target Selection (ii) Waiting (iii) Hunting (iv) Satisfaction. Each step of this process has certain pre-requisites to be fulfilled.

As with escape action, the predation also takes place in four stages: detection and target selection, waiting, hunting and satisfaction. The flow representation of this process is shown in Fig. 9. In the first stage, the predator takes notice of all the prey agents in its zone of detection *R*_*d*_, which, like in case of prey agents, is circular in nature (see Fig. 7(a)). For the sake of maintaining neutrality, the zone of detection for the predator is set to the same value as the zone of detection for the prey, and in the same vein, the predator also possesses a 360 vision. After detecting all the prey agents in this zone, the predator selects the prey agent that is the closest to it, as the target. This target selection mechanism, while simple in nature, is promising as shown by Demšar *et al*.^33^. After the particular agent has been marked, the predator waits for a certain time interval and meanwhile, keeps checking whether the target stays inside its *R*_*d*_. If the target is found to be within its detection zone for the wait time period, the predator moves on to the next stage, i.e. hunting. To this cause, the hunting force ${\overrightarrow{F}}_{h}$ acts on the predator (see Fig. 8(b_1_)) and this force, on being algebraically added to the other forces acting on it, results in a pursuit, in which the predator chases the target agent and kills it only when the target agent enters the predator’s sure-kill zone *R*_*k*_ (see Fig. 8(b_2_)). This sure-kill zone is adjusted so that the prey agent has to be in contact with the predator for it to be killed. However, as discussed earlier, only a negligible part of the killed agent is consumed. In case of hunting success, the predator moves to the next stage, i.e. satisfaction. This stage has been described by Demšar *et al*.^33^ as handling time, the time that lapses between the successful killing of the target prey and the subsequent target selection. Basically, this is the time when the predator neither searches for a prey nor hunts, i.e. it is satisfied with its foregoing forage. However, the alive prey agents being oblivious to this satiated state of mind of the predator still conduct escape manoeuvre during this time period. This behaviour has been incorporated into the model in order to add realism to the simulation.

The failure of a particular hunt can occur in two different ways:

1. If the target agent escapes the detection zone of the predator during the waiting stage.
2. If the predator pursues the target and is unable to capture it within a certain time limit referred to as time-out limit *t*_*To*_. This limit is taken to be many times the wait time, so as to allow the predator sufficient time to pursue the target.

Whenever any of these happens, the predator immediately goes back to the first stage, i.e. detection and target selection, as the satisfaction criteria remains unfulfilled. It is also possible that the target agent might approach the predator’s sure-kill zone due to the excessive force of the swarm propulsion, or rapid damping of the agitation stream^1^. In such a case, the predator kills the target prey and enters the satisfaction stage immediately, skipping any stages in between. The only criteria to be fulfilled in such a case is that the approaching prey agent must have been chosen as the target by the predator beforehand. This criteria can be detrimental to the hunting efficiency of the predator, but this phenomenon is backed by several experimental results and can be termed as a consequence of “predator confusion”^9^. In this case, the predator having locked down on the motion of a single prey agent is unable to focus on the motion of the other agents in its vicinity, and even though a prey agent approaches the predator, it still doesn’t get attacked as long as the predator is focused on the target agent. Hence, the other agents can flood and obscure the view of the predator resulting in hunting failure of the predator in any of the two ways mentioned above. A predator which fails in a such a fashion is termed as a confused predator^62^. The hunt mechanism has been covered in further details in Supplementary Video III.

### Simulation details

From a comparative study by Huth & Wissel^67,68^ and similar to the research by Kunz & Hemelrijk^69^, it is deduced that the collective motion of a system of self-propelled particles is virtually impervious to dimensionality. In the same vein, in the present work, simulations are performed in a 2-D square domain of side *L*. The wall is considered to be composed of fixed circular particles of radius *r*_*w*_. The prey agents are also simulated as disks of radius *r*. The predator has a similar morphology with radius *R*. The hunting and flight behaviours of the predator and the prey respectively kick in at time *t*_*h*_ after the start of the simulation. Waiting time and satisfaction time are set to *t*_*w*_ and *t*_*s*_ respectively, while time-out occurs at time *t*_*TO*_ after activation of the hunting force. For the prey agents, *r*_*d*_ is set to twenty times agent radius, and *r*_*l*_ is set to ten times agent radius (see Fig. 7(b)). For the predator agent, *R*_*d*_ is same as *r*_*d*_, *R*_*i*_ is ten times predator radius and *R*_*k*_ is set to 1.25 times the predator radius (see Fig. 7(a)). The values of the above parameters considered for the simulation are listed in Table 1.

**Table 1:** Details of parameters used in simulation (Note: The values of the parameters are presented in non-dimensional form).

Parameter	Value	Description
*n*	2808	Number of prey agents
*N*	1	Number of predator agents
*L*	1	Side length of the square 2D domain
*τ*	0–316	Simulation time
*χ*	121–6039	Co-ordination coefficient
*t*_*h*_	3.95	Time after which hunting and escape activates
**ℵ**	0.0625–1.250	Ratio of escape to hunting force
*r*	0.0625	Radius of prey agent
*R*	0.25	Radius of predator agent
*r*_*w*_	0.0625	Radius of wall particle
*r*_*d*_	20*r*	Radius of prey’s detection zone
*r*_*i*_	10*r*	Radius of prey’s influence zone
*R*_*d*_	5*R*	Radius of predator’s detection zone
*R*_*k*_	1.25*R*	Radius of predator’s sure-kill zone
*R*_*i*_	10*R*	Radius of predator’s influence zone
*t*_*w*_	0.08	Time period of waiting stage
*t*_*s*_	0.08	Time period of satisfaction stage
*t*_*TO*_	0.32	Time-out limit

The total force on any agent is first divided by its mass and then integrated using Verlet-Velocity algorithm^70^ to find velocity and position. Due to the high computational requirement of updating forces of such a large number of particles at each time step, a linked list algorithm is built into the code^71^. Due to the nature of this problem, there exists an initial position dependency with regard to the predator. Hence, twenty simulations have been done for any and all cases presented here. The initial position of the predator has been altered in all the cases and all the results obtained are averaged over all twenty trials, unless stated otherwise.

## Supplementary information

Supplementary Video I: Collective motion regimes for a non-consuming predator

Supplementary Video II: Fluctuations in temporal polarization

Supplementary Video III: Cycle of predation

Supplementary Video IV: Collective motion regimes for a consuming predator

Supplementary Information

## Data Availability

The datasets generated during and/or analysed during the current study are available from the corresponding author on reasonable request.
