# Supplementary material for: Confined System Analysis of a Predator-Prey Minimalistic Model
Source: Sci Rep. 2019 Aug 2;9:11258. doi: 10.1038/s41598-019-47603-9 (PMC6677773; doi:10.1038/s41598-019-47603-9)
Supplement: Supplementary file 5 — Supplementary Information [file 41598_2019_47603_MOESM5_ESM.pdf]

Confined System Analysis of a Predator-Prey Minimalistic Model

Siddhant Mohapatra[1] **and Pallab Sinha Mahapatra**[2,*]

[1]Department of Mechanical Engineering, National Institute of Technology Silchar, Silchar, India
[2]Department of Mechanical Engineering, Indian Institute of Technology Madras, Chennai, India
[*]Corresponding author: pallab@iitm.ac.in

Supplementary Information I: Effect of confinement

Simulations are run separately on a group of prey agents in presence of a singular predator in a closed as well as an open domain and the results were compared. Figure 1(a) shows the confined case and (b) shows the unconfined case. Three regime-specific co-ordination co-efficient χ values are chosen. The improvement in collective behaviour with increase in χ is observed in both the cases. The number of dead prey at the end of the simulation time N_d is employed as the parameter for comparison. A significant difference in the value of N_d is perceived in between the two cases. For instance, at $\chi = 1812$, in case of the closed domain, 33.19% of the prey are killed by the end of the simulation time, while in case of the open domain, the killed prey constitute a measly 2.24% of the entire prey populace. This is due to the difference in space available to conduct escape manoeuvres successfully. In the closed domain, the prey agents try to move away from the predator using their escape drive. But the walls provide an obstruction to the successful evasion of the prey and the front line agents of the fleeing flock having collided with the wall, enter a temporary confusion period, resulting in disorientation of the flock. This misalignment lasts for a short time, after which the prey re-oriented themselves by means of the co-ordination force and resume the escape manoeuvre. However, this short duration gives the predator an edge in hunting the confused prey. On the other hand, in case of the open domain, the prey move further away from the predator as there is no barrier to their motion. The flock is able to easily carry out the escape tactic. This can be seen in Fig. 1 for any of the listed χ values. Another reason of the low killing in the open domain relates to the disposition of the predator defined by us. Our predator is set to chase and kill the nearest prey. Once, the predator starts chasing a particular section of the prey, the other sections get farther from the predator as time passes. So the predator is focused only on the prey nearby it and doesn't pursue the prey agents far away. The predator is also unable to take sharp turns in its motion, unlike the prey which can route themselves in such a manner due to the nature of the co-ordination force and by virtue of their small size.

These simulations have been run after tweaking our original code based on a confined domain. Adding some restraining forces as in the case of Vabø & Nøttestad[1] would restrict the single-headed motion of the predator and contribute to improving the hunt behaviour. The parametric data considered for these simulations are listed in Table 1.

Table 1. Details of parameters used in confinement comparison simulation (Note: The values of the parameters are presented in non-dimensional form).

Parameter	Value	Description
n	2808	Number of prey agents
N	1	Number of predator agents
L	1	Side length of the square 2D domain
τ	0-79	Simulation time
χ	1812-6039	Co-ordination coefficient
t_h	0.79	Time after which hunting and escape activates
$\aleph$	0.0625	Ratio of escape to hunting force
r	0.0625	Radius of prey agent
R	0.25	Radius of predator agent
r_w	0.0625	Radius of wall particle
r_d	$20r$	Radius of prey's detection zone
r_i	$20r$	Radius of prey's influence zone
R_d	$5R$	Radius of predator's detection zone
R_k	$1.25R$	Radius of predator's sure-kill zone
R_i	$20R$	Radius of predator's influence zone
t_w	0.08	Time period of waiting stage
t_s	0.08	Time period of satisfaction stage
t_{TO}	0.32	Time-out limit

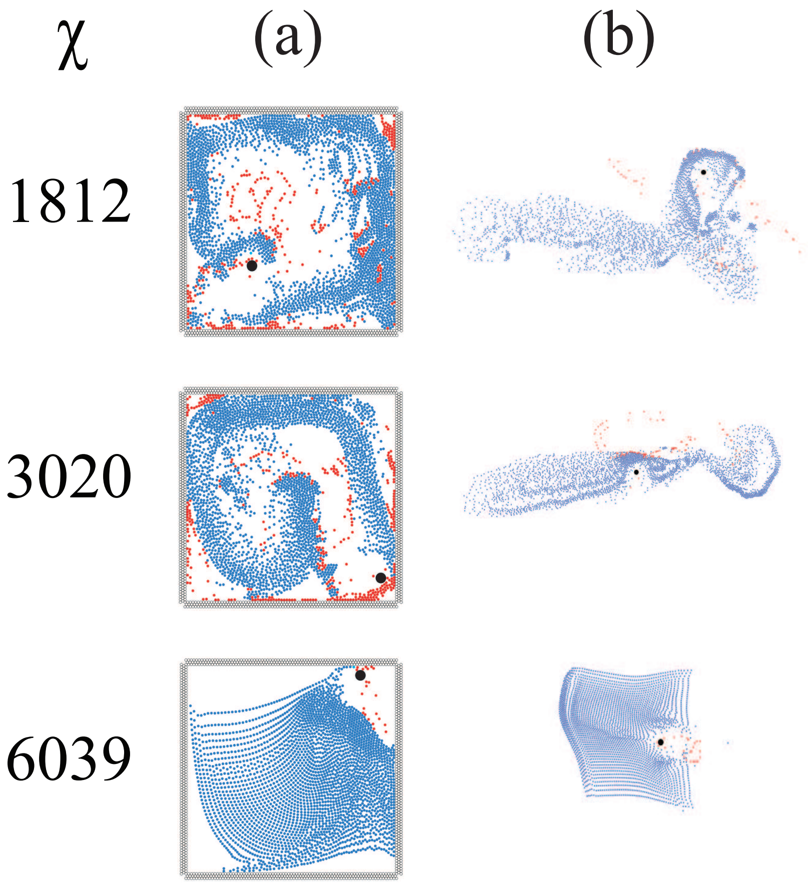

Figure 1. (Color online) Snapshots from the simulations comparing (a) confined and (b) unconfined domains are portrayed for different χ values. The number of deaths among the prey is clearly higher in case of the confined domain. This is due to the restraints enacted on the evading prey by the walls of the confinement, which causes a transient confusion amongst the prey, giving the predator ample opportunity to hunt. However, in the open domain, the prey agents are easily able to elude the predator. The number of deaths in the open domain is found to decrease as χ increases, a trend also observed in the confined case. (Note: Figures are not in same scale. Blue, red and black represents the live prey, dead prey and the predator respectively).

Supplementary Information II: Statistical significance of data

To understand the statistical trend of the data used in the plots, a few standard statistical techniques have been implemented. The variation of temporally averaged order parameter Φ is plotted for increasing χ values are shown in Fig. 2. The Pearson correlation or the "product moment correlation coefficient (PMCC)" of the data is $r = 0.82$. This signifies that the Φ and χ are linearly related on a straight ascending line. The same relationship can also be seen from the regression line of Fig. 2.

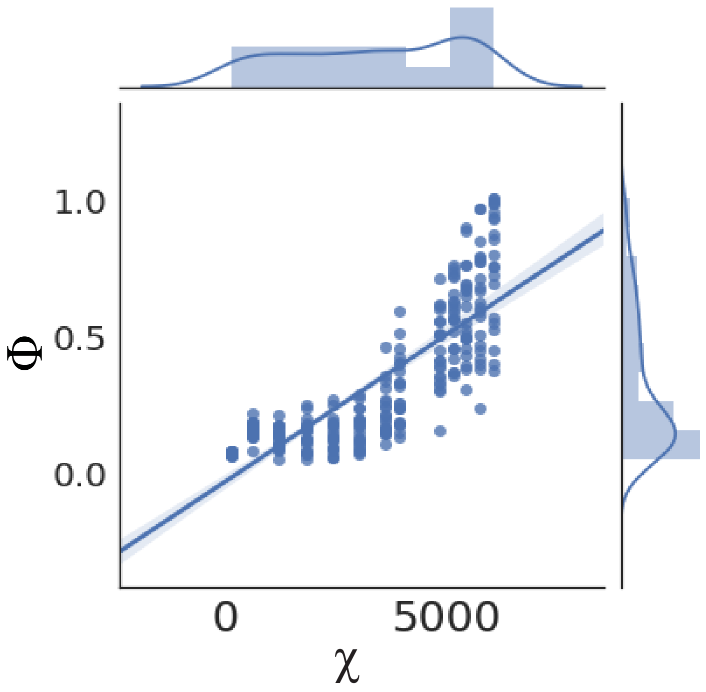

Figure 2. (Color online) The temporally averaged order parameter Φ is plotted for increasing χ values. The relationship of this bivariate system is shown along with the 1D profiles of the variables parallel to the respective axis. The linear regression is shown using the solid line.

In Fig. 3, the survival rate of the prey N_l is plotted with the increase of the non-dimensional parameter $\aleph$ (the ratio of escape to hunting force) for different χ. It can be seen from Fig. 3a, the regression line has an increasing trend with $r = 0.99$. With the increase of χ, the values of r decreases, for instance, at $\chi = 3020$ the value of r is 0.84 whereas, at $\chi = 6039$ the value of r is 0.71. The slow decrease of r suggests a slight departure from linear correlation between N_l and $\aleph$.

For further quantification, a Bayesian single-level linear regression model is used to see how the outcome variable (N_l) is related to the linear relation of $\aleph$. First the single level regression model is specified as $y_i \sim Normal(\beta_0 + \beta_1 x_i, \sigma^2)$[2]. Where, the outcome is y_i, the predictor variable is x_i, β_0 is the intercept, coefficient is β_1 and σ^2 is the variance. Markov chain Monte Carlo (MCMC) based *stan_glm* function in *rstanarm* is used to fit Bayesian linear regression model. Normal distributions for the intercept and coefficients are set as priors for the analysis. For $\chi = 121$, the prior used for the intercept has location 50 and standard deviation 100 and for the coefficient it is 0 and 1 respectively. The posterior summary statistics for $\chi = 121$ is shown in Table 2. It can be seen from Table 2 that, for the intercept (β_0) the mean of the posterior distribution is 1288.0, with 95% Posterior intervals ($PI = [1242.2, 1331.9]$). Similarly, Table 3 and Table 5 represents the posterior summary statistics for $\chi = 604$ and $\chi = 6039$ respectively.

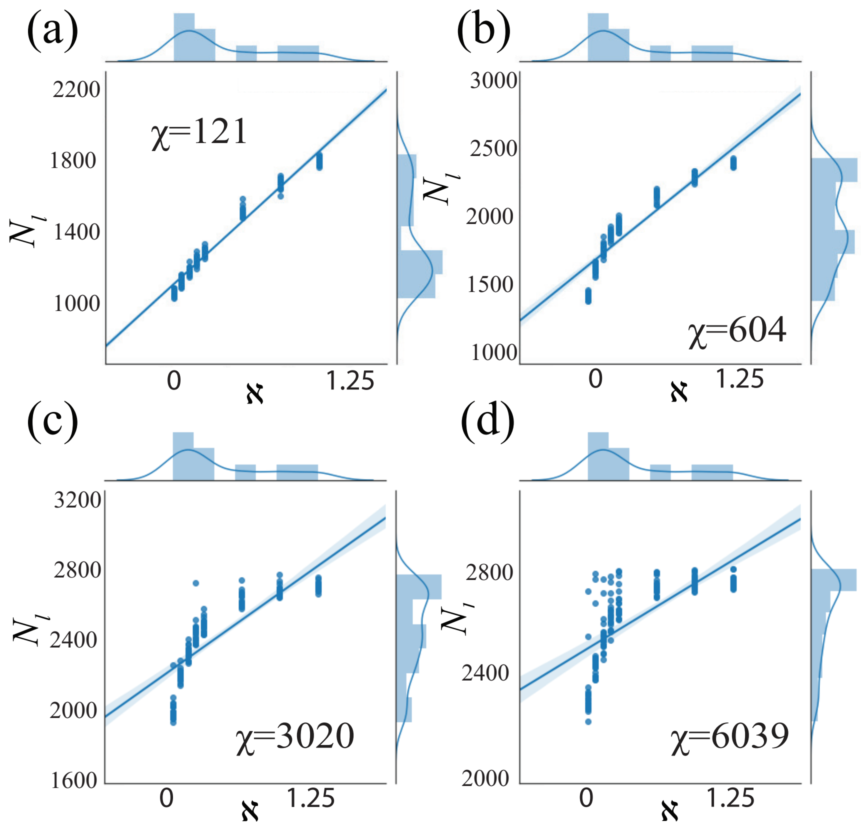

Figure 3. (Color online) The survival rate of the prey N_l is plotted for increasing ℵ (the ratio of escape to hunting force) values for different χ. The relationship of this bivariate system is shown along with the 1D profiles of the variables parallel to the respective axis. The linear regression is shown using the solid line.

Table 2. Posterior summary statistics for $\chi = 121$ after the Bayesian linear regression fit.

Parameter	Rhat	n_eff	mean	sd	2.5%	50%	97.5%
Intercept (β_0)	1.0	4949	1288.0	22.7	1242.2	1288.4	1331.9
Slope (β_1)	1.0	5272	0.8	1.0	-1.2	0.8	2.8
σ	1.0	5136	276.1	16.4	246.1	275.4	310.0

Table 3. Posterior summary statistics for $\chi = 604$ after the Bayesian linear regression fit.

Parameter	Rhat	n_eff	mean	sd	2.5%	50%	97.5%
Intercept (β_0)	1.0	3667	1793.8	30.4	1732.0	1794.4	1852.7
Slope (β_1)	1.0	5122	0.7	1.0	-1.3	0.6	2.6
σ	1.0	3995	337.0	22.1	298.3	335.8	383.7

Table 4. Posterior summary statistics for $\chi = 3020$ after the Bayesian linear regression fit.

Parameter	Rhat	n_eff	mean	sd	2.5%	50%	97.5%
Intercept (β_0)	1.0	4246	2345.0	23.4	2297.0	2345.9	2388.7
Slope (β_1)	1.0	4477	0.8	1.0	-1.2	0.8	2.7
σ	1.0	4102	255.8	16.8	226.0	254.6	291.3

Table 5. Posterior summary statistics for $\chi = 6039$ after the Bayesian linear regression fit.

Parameter	Rhat	n_eff	mean	sd	2.5%	50%	97.5%
Intercept (β_0)	1.0	5075	2559.3	13.9	2531.0	2559.8	2585.5
Slope (β_1)	1.0	5327	1.1	1.0	-0.8	1.1	3.1
σ	1.0	5047	163.2	10.1	145.1	162.7	184.2

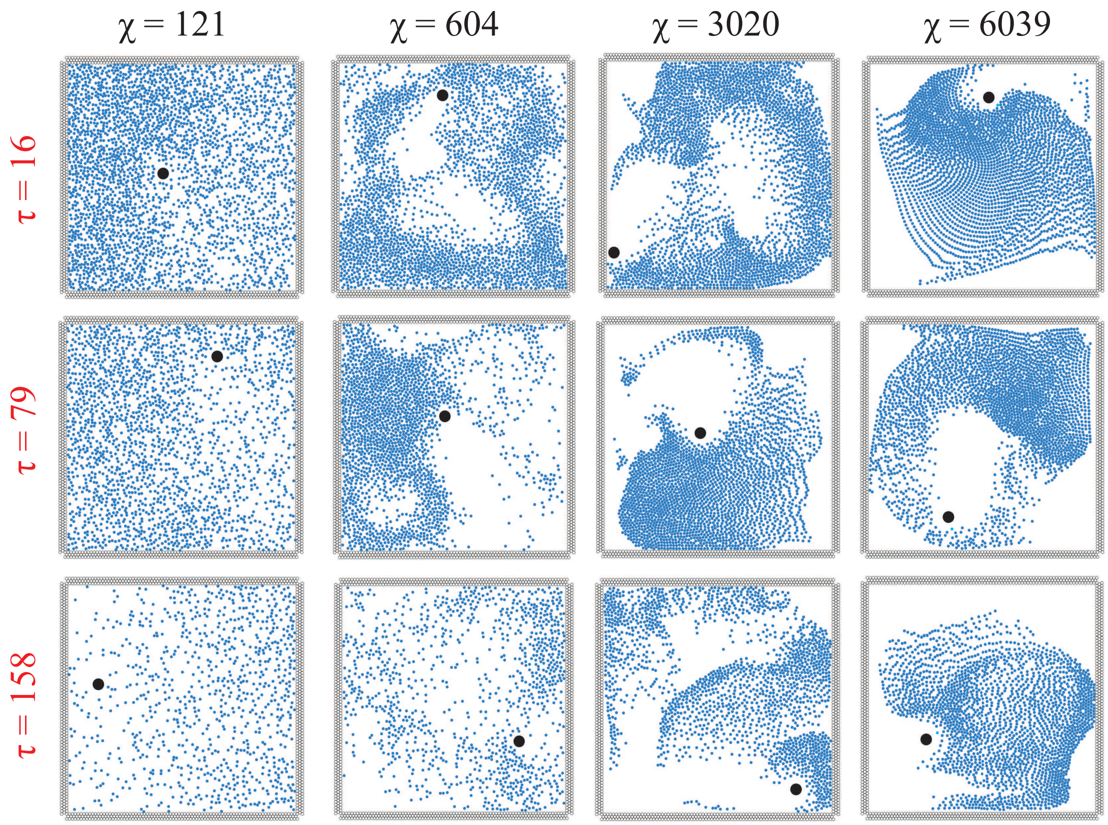

Figure 4. (Color online) The behaviour of agents is shown at different instances of non-dimensional time $\tau = t/\sqrt{\frac{L}{\beta}}$ under changing conditions of non-dimensional co-ordination co-efficient $\chi = \frac{C_v L\sqrt{L}}{m\sqrt{\beta}}$ in case of a consuming predator. The predator is coloured black, while live prey agents are coloured blue. At $\chi = 121$ and $\chi = 604$, thermal motion is observed, although traces of milling are evident in the latter. At $\chi = 3020$, a combination of distorted milling state with a filled core and oscillatory motion is observed. At $\chi = 6039$, the motion is predominantly oscillatory in nature. (Note: The initial position of the predator is $(0.93, 0.43)$ in a 1×1 domain. Images shown are representative of the usual trend observed over multiple initial positions of the predator. It is assumed that the predator's mass remains unchanged despite consumption).

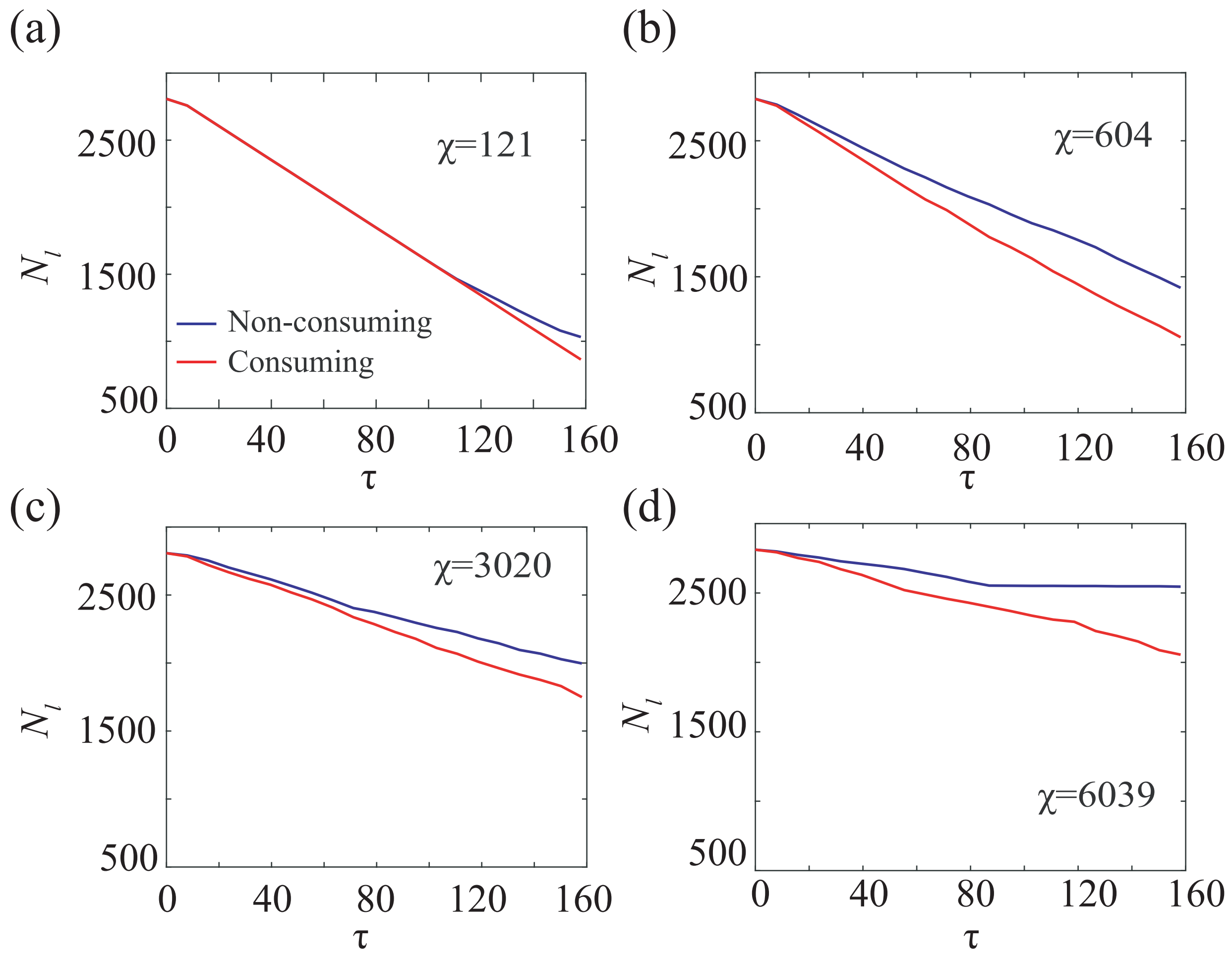

Figure 5. (Color online) The number of live prey agents N_l is plotted against non-dimensional time τ for four regime-specific values of non-dimensional co-ordination co-efficient χ. A comparison has been done between the case with the non-consuming predator and the consuming predator. It is observed that the rate of death of the prey agents is higher in case of consuming predator than that of the non-consuming case for all values of χ. A second observation is the increasing gulf between the rates of death in the two cases with increasing χ, the exception being $\chi = 604$. These features of the curve are direct consequences of the absence of dead prey agents in the domain. (Note: The initial position of the predator is $(0.93, 0.43)$ in a 1×1 domain).

Supplementary Information III: Effect of dead prey agents

The presence of the dead prey agents within the simulation bounds has an effect on the evasion success of the live prey and the pursuit success of the predator, as has been depicted in the manuscript. To highlight this effect, a few simulations have been run with the consideration of a consuming predator (i.e. the prey agents in this case disappear from the domain on death and are replaced by fluid medium). The mass and size of the predator, however, is assumed to be unchanged, for the sake of simplicity.

Figure 4 shows the behaviour of the agents at different instances of time under differing levels of co-ordination. At $\chi = 121$, thermal motion of the prey agents is observed through the time period of the simulation. At $\chi = 604$, thermal motion is still prevalent but traces of milling phase can be seen setting in. As a result, the range of motion of the prey agents is much larger compared to that of $\chi = 121$. However, as the time progresses, the system becomes increasingly sparser and thermal motion dominates over milling. At $\chi = 3020$, a transition between milling and dynamically parallel motion is observed. The oscillatory (i.e. dynamically parallel) behaviour is more prominent in the beginning of the simulation, but attenuates as time progresses. The milling phase witnessed in these simulations is distorted in nature and occurs with a dynamic core (that alternates between filling and emptying). At $\chi = 6039$, due to the high co-ordination level, the motion is purely oscillatory (dynamically parallel) throughout the simulation time. The predatory disturbances seem to play a more vital role in causing the regime based motion in these simulations as there is no spamming of dead agents in the predator's detection zone (explained in detail later). Supplementary Video IV can be referred for an illustration of the phenomenological regimes mentioned here.

The effect of the absence of the dead agents from the domain is further emphasized by Fig. 5. On comparing the non-consuming and the consuming predator cases on basis of the number of live prey agents N_l across the simulation time τ, there are two noteworthy inferences. The N_l versus τ curve is representative of the death rate of the prey agents throughout the simulation time. Comparing the death rates for the non-consuming and the consuming cases, it is clear from Fig. 5 that the death rate is higher in the case of the consuming predator as the slope of the non-consuming predator curve is higher than its counterpart. This is a result of improved predatory tactics, which can be attributed to higher freedom of movement of the predator due to the absence of the dead prey agents. The detection zone of the predator in the consuming case is free of dead agents and hence, can accommodate more number of live prey agents. From Fig. 5, the increasing difference between the death rates for the consuming and the non-consuming cases with increasing χ values is also discernible. This is due to the nature of the co-ordination force acting on the agents which is directly proportional to χ. For the predator, this co-ordination force acts like a friction force, inhibiting or slowing the predator down. In case of the non-consuming predator, due to this retardation on pursuit action, the predator is not able to exit the area immediately after killing and the detection zone of the predator gets swarmed by dead prey agents, decreasing the predator's effective detection zone. This, in turn, decreases the death rate of the prey agents. However, in the consuming predator case, because of the absence of the dead agents, the effective detection zone remains unaffected and the predator is able to pursue its next target more effectively, causing the death rate to increase at higher χ. Although the general trend shows an increasing gulf in the death rate comparison of the two cases, $\chi = 604$ is an exception. The difference in death rates at $\chi = 604$ is higher than that of $\chi = 3020$, which is against the general trend observed. This is due to the obstruction caused to the predator's pursuit motion by the mass of dead prey accumulated at the edges of the confinement in the non-consuming predator case. This decreases the number of deaths among the prey. On the other hand, at $\chi = 3020$, the accumulation is not sufficient to obstruct the motion of the predator, resulting in the disparity.

References

1. Vabø, R. & Nøttestad, L. An individual based model of fish school reactions: predicting antipredator behaviour as observed in nature. *Fish. oceanography* **6**, 155–171 (1997).

2. Muth, C., Oravecz, Z. & Gabry, J. User-friendly Bayesian regression modeling: A tutorial with rstanarm and shinystan. *Quant. methods for psychology* **14**, 99–119 (2018).
